# The effect of montelukast, a leukotriene receptor antagonist, on the acetic acid-induced model of colitis in rats: Involvement of NO-cGMP-K_ATP_ channels pathway

**DOI:** 10.3389/fphar.2022.1011141

**Published:** 2022-09-26

**Authors:** Behnam Ghorbanzadeh, Mohammad Amin Behmanesh, Roya Mahmoudinejad, Mehdi Zamaniyan, Shadi Ekhtiar, Yousef Paridar

**Affiliations:** ^1^ Department of Pharmacology, School of Medicine, Dezful University of Medical Sciences, Dezful, Iran; ^2^ Department of Histology, School of Medicine, Dezful University of Medical Sciences, Dezful, Iran; ^3^ Department of Internal Medicine, Dezful University of Medical Sciences, Dezful, Iran

**Keywords:** montelucast, nitric oxide, cGMP, cyclic guanosine monophosphate, KATP channels, rats, inflammatory bowel disease

## Abstract

Inflammatory bowel disease is a chronic autoimmune disorder that may involve entire gastrointestinal tract. The leukotrienes have a role as mediators in the pathophysiology of colitis. Here, we investigated the effect of a leukotriene receptor antagonist, montelukast, and also the role of the NO-cGMP-K_ATP_ channel pathway in acetic acid-induced colitis. Rectal administration of acetic acid (4%) was used for induction of colitis in rats. To investigate our hypothesis, the rats were intraperitoneally pre-treated with L-NAME (NOS inhibitor), L-arginine, sildenafil, methylene blue, glibenclamide, or diazoxide 15 min before treatment with montelukast (5–20 mg/kg, i. p.), for three consecutive days. Then, microscopic, macroscopic, and inflammatory parameters were evaluated. Montelukast reduced the microscopic and macroscopic damage induced by acetic acid. Montelukast also reduced the level of IL-1β and TNF-α. We also showed that the effects of montelukast were significantly attenuated by L-NAME, methylene blue (guanylate cyclase inhibitor), and an ATP-sensitive potassium channel blocker (glibenclamide). Also, the administration of L-arginine, sildenafil, and diazoxide before montelukast produced protective effect. In conclusion, the pathway of the NO-cGMP-KATP channel is involved in the protective effect of montelukast in acetic acid-induced colonic tissue damage.

## Introduction

Inflammatory bowel disease (IBD) which consists of ulcerative colitis and Crohn’s disease is inflammation of the alimentary tract. The industrialized world has a higher incidence of IBD, but it has risen in developing countries recently. Ulcerative colitis and Crohn’s disease are debilitating bowel disease that continues to be a serious health problem in the world such as colorectal cancer (Ng et al., 2018; Stidham and Higgins, 2018). The pathogenesis of ulcerative colitis and Crohn’s disease is not well established, but both disease results from leukocyte influx, and upregulation of inflammatory mediators (Hanauer, 2006; Torres et al., 2017). Also, the reactive oxygen species lead to the attraction of neutrophils at sites of inflammation, and cytokine releases such as IL-1β and TNF-α (Krieglstein et al., 2001). Aminosalicylates and glucocorticoids are the mainstays pharmacotherapies for ulcerative colitis and Crohn’s disease. Although the effective control of IBD is done by these agents, they produce many side effects. (Bryant et al., 2015). Therefore, research on the new therapeutic agents for the control of IBD could be helpful.

Nitric oxide (NO) is produced by NO synthase (NOS) from amino acid L-arginine. It has been shown that NO production seems to be beneficial in the animal models of experimental colitis, but the sustained increment of NO is detrimental (Kolios et al., 2004; Vallance et al., 2004). The activation of guanylate cyclase (GC) by NO leads to the production of cGMP. cGMP produces anti-inflammatory and regulatory effects including the inhibition of leucocyte adhesion and also platelet aggregation and vascular tone regulation. NO also could act as a superoxide scavenger to protect enzymes involved in prostaglandins and leukotrienes synthesis (Iwata et al., 2020). Moreover, pieces of evidence have shown that the activation of the K_ATP_ channels prevents the release of reactive oxygen species from mitochondria. So, potassium channel openers could produce anti-inflammatory effects (Wei et al., 2003).

Leukotrienes (LT) are produced by the 5-lipoxygenase enzyme from arachidonic acid. LTs as chemotactic and pro-inflammatory agents could increase microvascular permeability and have a role in inflammatory situations such as pain, rheumatoid arthritis, asthma, and ulcerative colitis (Ghorbanzadeh et al., 2016; Alizamani et al., 2021). Moreover, it has been reported that COX-2 expression and prostaglandin E production increased by leukotrienes D_4_ (LTD_4_) in the intestinal cells of humans and rats (Ohd et al., 2000).

Montelukast, a cysteinyl LT receptor antagonist (LTD_4_ antagonist), is used in the management of asthma. Furthermore, we revealed that montelukast exerts an anti-inflammatory effect in rats (Hemmati et al., 2013). Also, montelukast may have anti-inflammatory effects through interaction with the inflammatory transcription factor activation that is distinct from cysteinyl LT receptors antagonism (Weisberg, 2000; Wu et al., 2006). Moreover, it has been revealed that LT receptor antagonists could reduce the severity of colitis in different animal models (Nishikawa et al., 1995; Mahgoub et al., 2003). Recently, we observed that montelukast could modulate the NO-cGMP-K_ATP_ channel pathway in peripheral inflammatory pain models (Alizamani et al., 2021).

On these grounds, the present study aimed to investigate whether montelukast is able to reduce the severity of colitis induced by acetic acid in rats. Moreover, the probable involvement of the NO-cGMP-K_ATP_ channel pathway in the effect of montelukast was evaluated.

## Materials and methods

### Animals

Male Wistar rats (220 ± 20 g, 9–10 weeks) were obtained from the animal house of Dezful University of Medical Sciences. Animals were housed in laboratory conditions (relative humidity 60%–70%, temperature 24 ± 2°C, and 12-h light/dark cycle) with unrestricted access to food and water. Animals 24 h before the experiments fasted. The experimental procedures and animal handling were performed according to the ARRIVE guidelines and the National Research Council’s Guide for the Care and Use of Laboratory Animals. All experimental procedures were approved by the Animal Care Committee at Dezful University of Medical Sciences (IR.DUMS.REC.1400.012). Animals were randomly allocated into 17 study groups, by six

rats in each group Table 1. Total number of rats used in this study were 102. All efforts were made to minimize the number of animals used and their suffering.

**TABLE 1:** Treatment groups animals were allocated into 17 study groups, by six rats in each group.


Group 1	Sham group: without colitis induction + 0.9% normal saline solution/day
Group 2	Colitis group: 1 ml acetic acid 4% (rectal) + 0.9% normal saline solution/day
Group 3–5	Colitis + montelukast 5, 10 or 20 mg/kg/day
Group 6–7	Colitis + L-Arg 750 mg/kg/day + saline solution/day
	Colitis + L-Arg 750 mg/kg/day + montelukast 20 mg/kg/day
Group 8–9	Colitis + L-NAME 10 mg/kg/day + saline solution/day
	Colitis + L-NAME 10 mg/kg/day + montelukast 20 mg/kg/day
Group 10–11	Colitis + MB 20 mg/kg/day + saline solution/day
	Colitis + MB 20 mg/kg/day + montelukast 20 mg/kg/day
Group 12–13	Colitis + Sildenafil 5 mg/kg/day + saline solution/day
	Colitis + Sildenafil 5 mg/kg/day + montelukast 20 mg/kg/day
Group 14–15	Colitis + Diazoxide 3 mg/kg/day + saline solution/day
	Colitis + Diazoxide 3 mg/kg/day + montelukast 20 mg/kg/day
Group 16–17	Colitis + Glibenclamide 1 mg/kg/day + saline solution/day
	Colitis + Glibenclamide 1 mg/kg/day + montelukast 20 mg/kg/day

### Drugs

L-arginine hydrochloride (NO synthase substrate), L-NAME (non-selective inhibitor of NO synthase), and diazoxide (an ATP-sensitive K channel opener) were obtained from Sigma-Aldrich (St. Louis, Missouri, United States). Montelukast, sildenafil, and glibenclamide (ATP-sensitive K channel inhibitor) were kindly donated by Sobhan Pharmaceutical Co. (Tehran, Iran). Methylene blue (MB; guanylate cyclase inhibitor) was obtained from Merck. The used drugs were dissolved or suspended in normal saline (0.9% NaCl) and injected in a volume of 10 ml/kg. The selection of doses and route of administration were based on previous studies and also experiments in our laboratory (Mansouri et al., 2018; Naserzadeh et al., 2019; Dejban et al., 2020; Ajaman et al., 2021).

### Experimental colitis induction

Colitis in rats was induced according to MacPherson and Pfeiffer (MacPherson and Pfeiffer, 1978). Animals were anesthetized with ether slightly, and then an 8 cm long plastic catheter was inserted into the rat colon. Then, acetic acid 4% (1 ml) was administered rectally. For the prevention of acetic acid leakage, animals were maintained upside down for 1 min.

### Treatment protocols

One hour after administration of acetic acid (first day), single dose of montelukast (5, 10, and 20 mg/kg, i. p.) was injected and continued for another two consecutive days (days 2 and 3). On the fourth day, rats were sacrificed via the ether. After shaving the skin of the abdomen, it is incised by a surgical section. Then, the last 8 cm of the colon was excised, opened longitudinally, and washed. Finally, the colon was divided into two parts: one part was embedded in formalin 10% solution for histopathological assessment, and the other part for biochemical evaluations was frozen at -80°C.

To study the role of NO in the effect of montelukast, a NO synthase inhibitor (L-NAME, 10 mg/kg) or a NO synthase substrate (L-arginine, 750 mg/kg, i. p.) were also applied for 3 days, 15 min before the treatment with montelukast (20 mg/kg, i. p.). Moreover, L-NAME (10 mg/kg, i. p.) or L-arginine (750 mg/kg, i. p.) alone was also administered followed by saline injection (without montelukast) for 3 days after rectal administration of acetic acid.

To study the involvement of cGMP in the action of montelukast, the inhibitor of the soluble guanylate cyclase (MB, 20 mg/kg) or a PDE-5 inhibitor (sildenafil, 5 mg/kg, i. p.), was also applied for 3 days, 15 min before the treatment with an effective dose of montelukast (20 mg/kg, i. p.). These drugs are also applied alone as described above.

To study the involvement of K_ATP_ channels in the action of montelukast, the inhibitor of K_ATP_ channels (glibenclamide, 1 mg/kg) or a K_ATP_ channels opener (diazoxide, 3 mg/kg, i.p.), was also applied for 3 days, 15 min before the effective dose of montelukast (20 mg/kg, i. p.). These drugs are also applied alone as described above.

### Evaluation of colonic damage

The macroscopic scores were assessed under a magnifying glass by an observer based on the following criteria: 0, without macroscopic changes; 1, only presence of mucosal erythema; 2, presence of mild mucosal edema, slight erosion, or slight bleeding; 3, presence of moderate edema, erosions, or bleeding ulcers; and 4, presence of severe edema, ulceration, and necrosis of colonic tissue (Deshmukh et al., 2010).

### Body weight measurement

Rats were weighed daily in all groups to consider the body weight changes during the study.

### Histopathological assessment

For histopathological evaluation, a sample of the colon was harvested and fixed in a 10% formalin solution. Then, samples were embedded in paraffin and sectioned at 4 μm. Then, the sections were stained with hematoxylin and eosin and examined under a light microscope.

### Investigation of cytokine levels

The pro-inflammatory cytokines including tumor necrosis factor (TNF-α) and interleukin (IL-1β) levels were measured using an enzyme-linked immunosorbent assay (ELISA) from Karmania Parsgen (Kerman, Iran) by following the manufacturer’s instructions. ELISA results were expressed as picograms per gram of tissue (pg/g tissue).

### Statistical analysis

Data are expressed as the mean ± standard errors of the mean (SEM) of six animals per group. The dose of montelukast reducing the macroscopic score in colon tissue by 50% relative to the control (ED_50_) was measured by linear regression from individual experiments using GraphPad software (GraphPad Prism 7.05, San Diego, CA, United States). The reducing the macroscopic score in colon tissue was expressed as percent of maximum possible effect (%MPE) that was calculated by the following equation: %MPE = [100 × (mean of macroscopic score in control group-mean of macroscopic score in drug(s)-treated group)]/mean of macroscopic score in control group. Normal distribution of data was determined via Kolmogorov–Smirnov test. Analysis of Variance (ANOVA) followed by Tukey’s multiple comparisons was used to compare means. *p* < 0.05 was indicated as statistically significant.

## Results

### Effects of montelukast on macroscopic appearance

**FIGURE 1:**
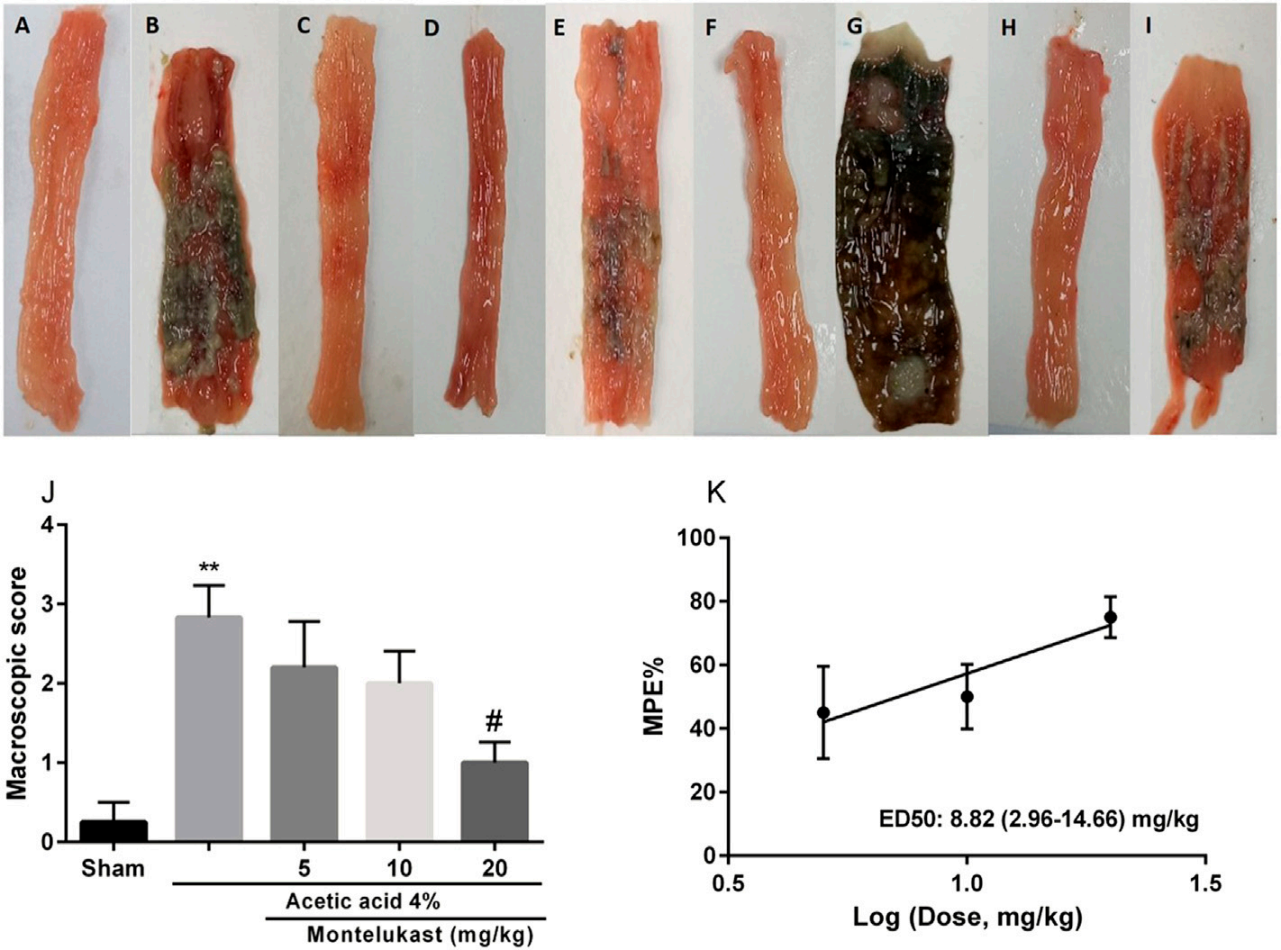
Effect of montelukast (Mon) on the macroscopic score of colon tissue **(A)** Sham; **(B)** acetic acid group **(C)** Mon (20 mg/kg) **(D)** Mon 20 + L-arg; **(E)** Mon 20 + L-NAME **(F)** Mon 20 + sildenafil (sil) **(G)** Mon 20 + Methylene blue (MB) **(H)** Mon 20 + diazoxide (Diaz) **(I)** Mon 20 + glibenclamide (Gli). Evaluation of the macroscopic scores and ED50 value (with 95% confidence limits) of Mon for the protective effect shown in panels **(J)** and **(K)**, respectively. The results are expressed as the means ± SEM of six rats per group. **p < 0.01, when compared to the Sham group; ^##^ *p* < 0.01 when compared to the acetic acid group. The statistical analysis was performed by ANOVA followed by Tukey’s *post hoc* test. %MPE: Maximal possible effect.

As shown in Figure 1 in the Sham group (normal saline administered rectally) no colonic lesion was observed. In the acetic acid group, tissue showed severe tissue damage significantly compared with the Sham group (*p* < 0.01). However, with the administration of montelukast (20 mg/kg, i.p.), colonic tissue damage was improved significantly, and the macroscopic score decreased remarkably [F (4,25) = 8.67, *p* < 0.001]. ED_50_ values (with 95% confidence interval limits) for the effect of montelukast was 8.81 (2.96–14.66) mg/kg.

### Role of NO in the protective effect of montelukast

**FIGURE 2:**
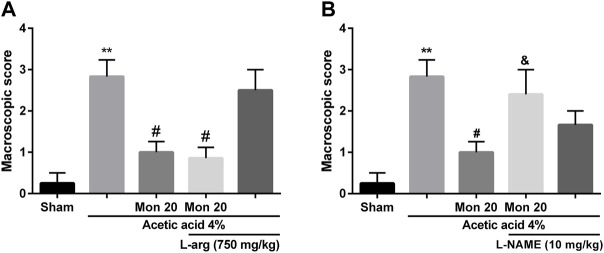
Evaluation of the involvement of the NO in the protective effect of montelukast (Mon, 20 mg/kg) on the macroscopic score of colon tissue. The effect of pre-treatment with L-arginine (L-arg, 750 mg/kg, (I) p.) and L-NAME (10 mg/kg, (I) p.) on the effect of Mon is shown in panels **(A)** and **(B)**, respectively. The results are expressed as the means ± SEM of six rats per group. **p < 0.01, when compared to the Sham group; ^#^ *p* < 0.05 when compared to the acetic acid group; ^&^ *p* < 0.05, when compared to the Mon-treated group. The statistical analysis was performed by ANOVA followed by Tukey’s *post hoc* test.

To assess the involvement of NO in the effects of montelukast, rats were pre-treated with an inhibitor of the NO synthase (L-NAME) or a substrate for NO synthase (L-arginine). Figure 2 shows that L-arginine improves the protective effect of montelukast when administered before it [F (4,25) = 10.35, *p* < 0.01]. While administration of L-NAME. attenuated the effect of montelukast in both macroscopic and microscopic evaluations significantly [F (4,25) = 6.61, *p* < 0.01, Figures 2, 6]. Additionally, administration of L-arginine or L-NAME alone was ineffective.

### Role of cGMP in the protective effect of montelukast

**FIGURE 3:**
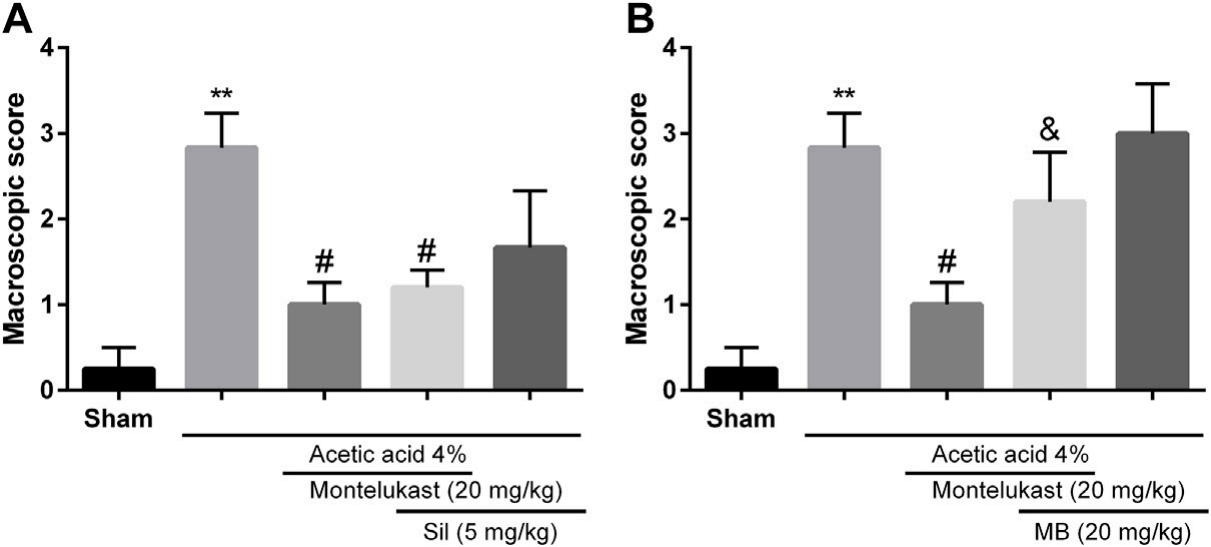
Evaluation of the involvement of the cGMP in the protective effect of montelukast (Mon, 20 mg/kg) on the macroscopic score of colon tissue. The effect of pre-treatment with sildenafil (Sil, 5 mg/kg, (I) p.) and Methylene blue (MB, 20 mg/kg, (I) p.) on the effect of Mon is shown in panels **(A)** and **(B)**, respectively. The results are expressed as the means ± SEM of six rats per group. **p < 0.01, when compared to the Sham group; ^#^p < 0.05 when compared to the acetic acid group; ^&^p < 0.05, when compared to the Mon-treated group. The statistical analysis was performed by ANOVA followed by Tukey’s post hoc test.

In the present work, we aimed to investigate whether cGMP participated in the effect of montelukast in acetic acid-induced colitis. Results show administration of sildenafil in combination with montelukast produced a protective effect in colitis induced by acetic acid [F (4,25) = 7.23, *p* < 0.001]. While, administration of an inhibitor of cGMP formation, methylene blue, attenuated the effect of montelukast in both macroscopic and microscopic evaluations significantly [F (4,25) = 8.12, *p* < 0.001, Figures 3, 6]. Additionally, methylene blue and sildenafil were ineffective when used alone.

### Role of ATP-sensitive potassium channels (K_ATP_) in the effect of montelukast

**FIGURE 4:**
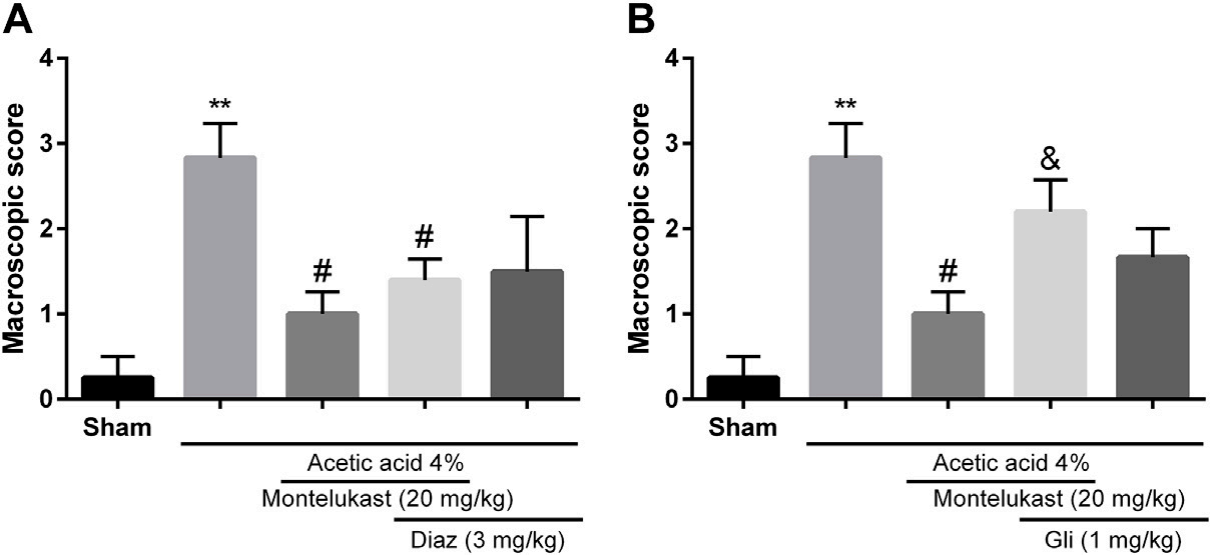
Evaluation of the involvement of the KATP channel in the protective effect of montelukast (Mon, 20 mg/kg) on the macroscopic score of colon tissue. The effect of pre-treatment with diazoxide (Diaz, 3 mg/kg, (I) p.) and glibenclamide (Gli, 20 mg/kg, (I) p.) on the effect of Mon is shown in panels **(A)** and **(B)**, respectively. The results are expressed as the means ± SEM of six rats per group. **p < 0.01, when compared to the Sham group; ^#^p < 0.05 when compared to the acetic acid group; ^&^p < 0.05, when compared to the Mon-treated group. The statistical analysis was performed by ANOVA followed by Tukey’s post hoc test.

To assess the contribution of K_ATP_ channels in the effects of montelukast, glibenclamide or diazoxide was administered before montelukast. Figure 4 shows that administration of diazoxide in combination with montelukast produced a protective effect in colitis induced by acetic acid [F (4,25) = 8.71, *p* < 0.001]. While, the administration of glibenclamide, a K_ATP_ channels inhibitor, attenuated the effect of montelukast in both macroscopic and microscopic evaluations significantly [F (4,25) = 6.69, *p* = 0.0014, Figures 4, 6]. Additionally, glibenclamide and diazoxide were ineffective when used alone.

### Evaluation of the body weight

**FIGURE 5:**
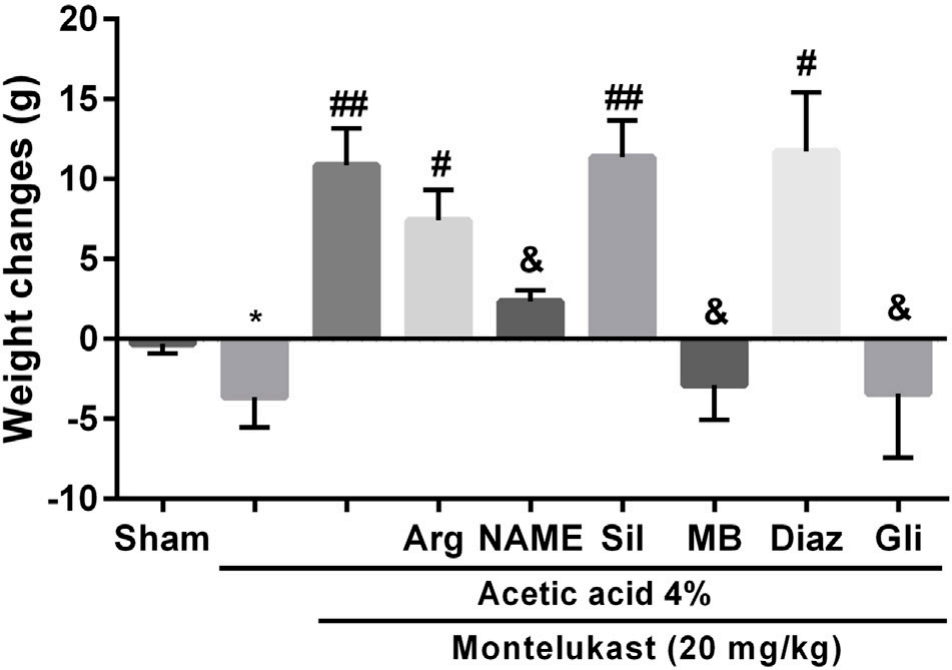
The involvement of NO-cGMP-KATP channels on the effect of montelukast on the body weight reduction induced by acetic acid. A significant decrease in body weight in acetic acid-treated rats was observed. *p < 0.01, when compared to the Sham group; ^##^ *p* < 0.05 when compared to the acetic acid group; ^&^ *p* < 0.05, when compared to the Mon-treated group. The statistical analysis was performed by ANOVA followed by Tukey’s post hoc test.

On the fourth day, acetic acid-induced colitis caused significant weight loss compared with the Sham group. However, after the administration of montelukast (5–20 mg/kg, i.p.), animals showed significant weight gain. However, pre-treatment with L-NAME, MB, and glibenclamide 15 min before montelukast (20 mg/kg, i.p.) caused significant weight loss in comparison with the montelukast group [F (8,45) = 8.97, *p* < 0.0001, Figure 5]. In addition, intraperitoneal administration of L-arg (750 mg/kg), sildenafil (5 mg/kg), and diazoxide (3 mg/ kg) before the administration of montelukast improved the effect of montelukast on weight gain.

### Effects of montelukast on histopathologic features

**FIGURE 6:**
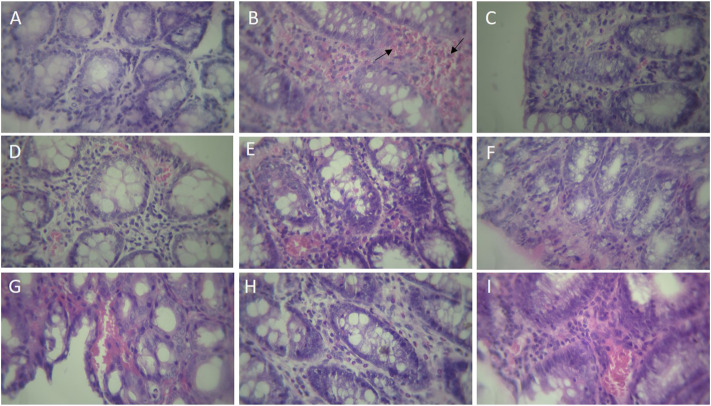
Histopathological examination of rat’s colonic tissue (magnification, × 400). Photomicrographs of hematoxylin and eosin-stained paraffin sections of rat colonic tissues **(A)** Sham; **(B)** acetic acid group, which shows ulceration with necrosis (arrow) of the entire wall or part of it, transmural inflammation, and irregular villous mucosal surface **(C)** montelukast (20 mg/kg), which attenuates the extent and severity of the histological signs of cell damage **(D)** Mon 20 + L-arg; **(E)** Mon 20 + L-NAME **(F)** Mon 20 + sildenafil (sil) **(G)** Mon 20 + Methylene blue (MB) **(H)** Mon 20 + diazoxide (Diaz) **(I)** Mon 20 + glibenclamide (Gli).

The histologic features of each group were shown in Figure 6. In the Sham group, crypt damage and inflammation were not observed in colon tissue. Colitis induced by acetic acid showed destruction of epithelium and crypts, and severe infiltration of inflammatory cells. However, montelukast (20 mg/kg, i.p.) improved the crypt damages and inflammation remarkably. Furthermore, intraperitoneal administration of L-NAME (10 mg/kg), MB (20 mg/kg), and glibenclamide (1 mg/ kg) before montelukast (20 mg/kg, i.p.) attenuated the effects produced by montelukast. In addition, intraperitoneal administration L-arg (750 mg/kg), sildenafil (5 mg/kg), and diazoxide (3 mg/kg) before the administration of montelukast (20 mg/kg, i.p.) improved protective effect of montelukast. However, administration of these modulators alone in colitis groups showed inflammation and crypt damage (data not shown).

### Effect of montelukast on TNF-α and IL-1β in colonic injury by acetic acid

**FIGURE 7:**
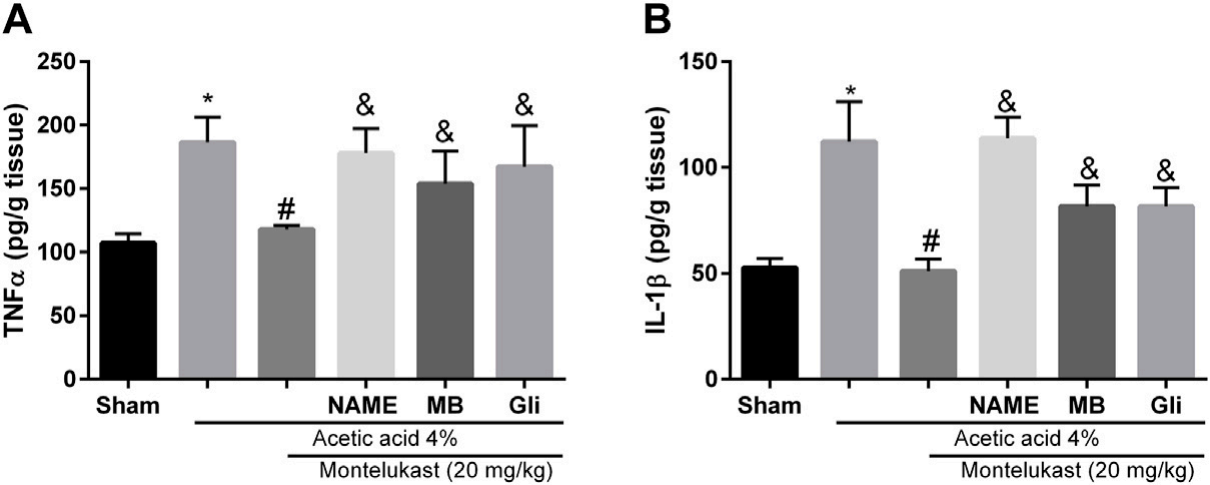
Montelukast (Mon) reduces TNF-α **(A)** and IL-1β **(B)** levels in colon tissue. L-NAME, methylene blue (MB), and glibenclamide (Gli) restore the TNF-α and IL-1β levels produced by Mon. *p < 0.05, when compared to the Sham group; ^#^ *p* < 0.05 when compared to the acetic acid group; ^&^ *p* < 0.05, when compared to the Mon-treated group. The statistical analysis was performed by ANOVA followed by Tukey’s *post hoc* test.

As shown in Figure 7, the level of these pro-inflammatory biomarkers in the group that received acetic acid was increased compared to the saline group significantly (*p* < 0.05). Administration of montelukast (20 mg/kg, i.p.) produced a significant attenuation (*p* < 0.05) in the levels of the TNF-α and IL-1β in acetic acid-induced colitis. This result shows that montelukast is capable of reducing the levels of TNF-α and IL-1β. Moreover, the effect of montelukast on IL-1β and TNF-α was attenuated by L-NAME, MB, and glibenclamide (*p* < 0.05). However, the effect of L-arginine, sildenafil, and diazoxide before montelukast was insignificant on TNF-α and IL-1β levels compared to the montelukast group (data not shown).

## Discussion

In the present work, we found that montelukast significantly inhibited colitis induced by acetic acid. Also, montelukast attenuated the levels of pro-inflammatory cytokines such as TNF-α and IL-1β in the colon tissue. Further investigation indicated that the pathway of the NO-cGMP-K_ATP_ channel mediates the protective effect of montelukast.

The exact causes of inflammatory bowel disease (IBD) are not well recognized, but several factors such as enhanced vascular permeability, neutrophils recruitment, and inflammatory mediators production are involved (Hanauer, 2006). Moreover, the elevation of cysteinyl LTs in mucosal layers is involved in ulcerative colitis and Crohn’s disease (Jupp et al., 2007). The LTs generated mainly by macrophages, mast cells, and eosinophils, and they are involved in stimulating the secretion of mucus and edema (Spada et al., 1986). They also cause the contraction of smooth muscle in the colon (Goldenberg and Subers, 1982). These effects result in mucosal ulceration, diarrhea, rectal bleeding, and weight loss (Ungaro et al., 2017). In the present study, for induction of an experimental model of IBD in animals, colitis was induced by rectal administration of acetic acid in rats. Here, we found that montelukast attenuated colitis induced by acetic acid. Administration of montelukast improved macroscopic scores and pathological damage in the colon significantly, and also attenuated the inflammatory cytokine elevation. In this regard, it has been shown that pranlukast, a LT receptor antagonist, decreases colitis induced by TNBS (Nishikawa et al., 1995). In addition, zafirlukast, a LT receptor antagonist, has a prophylactic effect in colitis induced by acetic acid (Mahgoub et al., 2003). Moreover, montelukast (20 mg/kg) improved the weight loss seen in the colitis group indicating a beneficial effect. In this regard, Holma et al. (2007) showed that montelukast inhibited the reduction of weight gain seen in rats with colitis induced by dextran sulphate sodium (Holma et al., 2007).

Nitric oxide (NO) has many physiological roles (Luo and Cizkova, 2000). The enzyme NO synthase (NOS) produced NO from the L-arginine substrate. NO can induce some effects in cells directed by itself or by the production of cGMP through activation of guanylate cyclase (GC). It has been shown that cGMP or NO can activate ATP-sensitive K channels (K_ATP_). On the other hand, it has been shown that LTC_4_, LTD_4,_ and LTB_4_, could affect inflammatory cells by surface receptors to release NO (Lärfars et al., 1999). It has been reported that NO could produce mucosal defense through influences the mucus secretion, blood flow, and neutrophil adhesion (Wallace and Miller, 2000). Moreover, the review of the literature confirms that NO is involved in the pathogenesis of IBD. In this regard, it has been shown that NO which is synthesized by NOS could have some protective effects on processes of inflammation in colitis. Also, severe damages were observed after the loss of each isoform of NOSs (Roediger et al., 1986), although controversial results have also been reported (Rachmilewitz et al., 1995). In this study, we observed that L-arginine (a NO precursor) potentiated the montelukast protection effect. Further, we evaluated the synthesis of NO by NO synthase (NOS), to recognize if the effect of montelukast is based on the activation of the L-arginine-NO route. Accordingly, pre-treatment with L-NAME (a non-selective NOS inhibitor) attenuated the protective effect of montelukast. So, the effect of montelukast against acetic acid-induced colitis was through activation of L-arginine-NO route. Hosoi et al. (2001) suggested that NO produces protective effects in the inflammation of colonic tissue (Hosoi et al., 2001). Previously, we indicated the participation of NO in antinociceptive effect of montelukast (Alizamani et al., 2021).

Next, we aimed to investigate the involvement of cyclic guanosine monophosphate (cGMP) in this effect. Levels of cGMP are increased by the action of NO on soluble GC. Cyclic GMP plays a pivotal role in the maintenance of mucosal homeostasis and regulation of intestinal inflammation (Harmel-Laws et al., 2013). In the present work, we showed that soluble GC inhibition by methylene blue (MB) attenuated the protective effects of montelukast against acetic acid-induced colitis. Moreover, co-administration of sildenafil, an inhibitor of PDE5, with montelukast produced protective effect in colitis induced by acetic acid. Our findings are compatible with those of Fakhfouri et al. (2012), who showed that pre-treatment with ODQ, an inhibitor of GC, antagonized the protective effect of sildenafil on colonic damage (Fakhfouri et al., 2012).

Evidence confirmed that NO and cGMP could activate some types of K channels such as K_ATP_ channels (Jeong et al., 2001). The modulation of cell membrane ion channels, especially potassium channels have a role in the mechanisms of the inflammation process in IBD (Magalhães et al., 2016). Previous investigations reported that activation of the mitochondrial K_ATP_ channels inhibits the release of reactive oxygen species; So it could be suggested that potassium channel openers such as diazoxide may have anti-inflammatory effects. Here, we have observed that K_ATP_ channels blockade with glibenclamide, inhibited the effect of montelukast against acetic acid-induced colitis. Moreover, co-administration of diazoxide, a potassium channel opener, with montelukast produced protective effect against acetic acid-induced colitis. In this regard, The anti-ulcer activity of some potassium channel openers like diazoxide has been proved (Toroudi et al., 1999). Also, the involvement of K_ATP_ channels in several models of colitis has been shown (Fakhfouri et al., 2012). Moreover, Daneshmand et al. (2011) reported that glibenclamide, a K_ATP_ channels blocker, worsens colitis in rats (Daneshmand et al., 2011).

The cytokines with pro-inflammatory properties such as TNF-α and IL-1β are increased during the experimental model of IBD (Ludwiczek et al., 2004). During colitis, TNF-α and IL-1β are secreted from monocytes and leukocytes (Shen et al., 2007). Accordingly, our study showed that rectal acetic acid administration produced colonic damage by elevation in the pro-inflammatory cytokine IL-1β and TNF-α levels. Further, it has been reported that NO inhibits the interactions between endothelial cells and inflammatory cells by decreasing the adhesion molecules. So, NO exerts tissue protective effect in process of inflammation (Kubes et al., 1994). Results obtained here revealed that these cytokines were lower in groups treated with montelukast significantly. Moreover, the effect of montelukast on TNF-α and IL-1β was antagonized by L-NAME, MB, and glibenclamide, suggesting the role of NO-cGMP-K_ATP_ channels in this effect. In a previous report, we indicated that montelukast exerts anti-inflammatory influence (Hemmati et al., 2013). Moreover, it has been indicated that many functions of NO such as vasodilation and consequently inhibition of edema, and also inflammatory cell adherence reversed by glibenclamide (Medeiros et al., 2009).

In conclusion, our investigation revealed that montelukast prevents colitis induced by rectal administration of acetic acid by reducing the inflammation, and the observed effect seems to be related, at least in part, to the modulation of the pathway of the NO-cGMP-K_ATP_ channel. So, montelukast may be a promising drug against IBD in a clinical setting.

## Data Availability

The original contributions presented in the study are included in the article/supplementary materials, further inquiries can be directed to the corresponding author.
